# Genome-Wide Identification of the HD-ZIP Transcription Factor Family in Maize and Functional Analysis of the Role of *ZmHD-ZIP23* in Seed Size

**DOI:** 10.3390/plants14162477

**Published:** 2025-08-10

**Authors:** Jinghua Zhang, Xuan Zhang, Mengru Liu, Yichen Jin, Qiaofeng Pai, Xiaolin Wu, Doudou Sun

**Affiliations:** 1 State Key Laboratory of High-Efficiency Production of Wheat-Maize Double Cropping, College of Life Sciences, Henan Agricultural University, Zhengzhou 450046, China; zhangjinghua@henau.edu.cn (J.Z.); zhangxuan0280@163.com (X.Z.); liumengru54@163.com (M.L.); jjyycc2002@163.com (Y.J.); paiqiaofeng2022@163.com (Q.P.); wuxiaolin@henau.edu.cn (X.W.); 2 The Shennong Laboratory, Institute of Cereal Crops, Henan Academy of Agricultural Sciences, Zhengzhou 450002, China; 3 Postdoctoral Station of Crop Science, Henan Agricultural University, Zhengzhou 450046, China

**Keywords:** cis-acting elements, gene expression, seed size, transcription factor, ZmHD-ZIP

## Abstract

HD-ZIP proteins (homeodomain–leucine zipper proteins) are a large family of plant-specific transcription factors that play crucial roles in regulating various physiological and developmental processes, including growth, differentiation, response to environmental stress, and reproductive development. Seed size is the main limiting factor affecting the yield of maize (*Zea mays*). However, the genome-wide identification and characterization of this family in maize and its biological functions in seed size have not been reported. Here, 61 *ZmHD-ZIP* genes were identified in the genome. Phylogenetic analysis of these *ZmHD-ZIP* genes revealed that they are clustered into four subfamilies: HD-ZIP I, HD-ZIP II, HD-ZIP III, and HD-ZIP IV. Domain analysis revealed that the distribution of these domains aligns perfectly with the subfamily classification criteria, with members of the same subfamily sharing similar domains. Cis-acting element analysis showed that the ZmHD-ZIP family genes are involved in the regulation of plant reproductive development. In addition, *ZmHD-ZIP23*-overexpressing Arabidopsis and maize had larger seed with increased grain length and heavier grain weight owing to bigger embryo and endosperm area. These findings could serve as a basis for future studies on the roles of *ZmHD-ZIP* genes in improving maize yield.

## 1. Introduction

Cereal crops such as maize (*Zea mays*), wheat (*Triticum aestivum*), and rice (*Oryza sativa*) are major sources of staple food, and their yield is closely related to plant growth and development [1]. Transcription factors play a crucial regulatory role throughout plant growth and development, including processes such as nutrient absorption, response to environmental signals, and adaptation to stress. Among them, HD-ZIP proteins, a unique class of transcription factors in plants, perform essential functions in plant growth, development, and stress responses. They are involved in processes like embryogenesis, vascular tissue formation, response to abiotic stress, and hormone signaling [2,3,4,5].

HD-ZIP proteins belong to the homeobox family and are a class of plant-specific transcription factors. They contain two important conserved elements: the homeodomain (HD) and the leucine zipper (LZ) domain. The HD domain consists of 60 or 61 conserved amino acids encoded by the *Homeobox* gene [6] and folds into a unique three-helix structure, where helices I and II are anti-parallel, and helix III is positioned perpendicular to helices I and II. Helix III corresponds to a DNA recognition region that enters the major groove of the DNA double helix, establishing specific contact with the minor groove at the unstructured N-terminus of helix I. This interaction forms a tight bond to the target DNA double helix in a helix–loop–helix (HLH) configuration [7]. In the LZ domain, leucine residues are repeated every seven amino acids, and the side chains of these leucine residues protrude and align neatly on the exterior of the polypeptide chain. This structural feature allows two HD-ZIP proteins to form a dimer through hydrophobic interactions [8]. The HD domain is responsible for DNA binding, while the LZ domain assists in dimer formation. Together, these domains function to recruit regulatory factors and regulate the expression of downstream genes.

Based on the sequence conservation of the HD and LZ domains, the presence of other conserved domains, gene structure, and biological functions, HD-ZIP proteins are classified into four subfamilies: HD-ZIP I, HD-ZIP II, HD-ZIP III, and HD-ZIP IV [2,7]. HD-ZIP proteins play a key regulatory role in plant growth and development, such as by regulating the angle and number of tillers, phytohormone synthesis and metabolism, leaf morphology, inflorescence, and seed development [9,10,11,12,13].

The tillering, tiller angle, and number of tillers in cereal plants determine, to some extent, crop yield. Optimizing these three traits to improve crop yield has long been a focus of crop breeding efforts. Recent large-scale transcriptome data analysis has revealed the core regulatory pathways involved in tiller angle formation, which primarily include two key genes: *HEAT STRESS TRANSCRIPTION FACTOR 2D* (*HSFA2D*) and *LAZY1* (*LA1*) [9]. The rice HD-ZIP II subfamily genes *Homeobox1* (*OsHOX1*) and *Homeobox28* (*OsHOX28*) regulate the distribution of auxin by inhibiting the HSFA2D-LA1 pathway and reducing the endogenous auxin content, thus controlling the size of the tiller angle [10]. During maize domestication and crop improvement, the inhibition of lateral branch growth has been an important trait selected for [11]. The maize HD-ZIP I subfamily gene *Grassy Tillers 1* (*ZmGT1*) regulates tillering by promoting axillary bud dormancy and inhibiting lateral branch elongation. Its expression is induced by shading and is controlled by another upstream tiller-regulating gene, *Teosinte Branched 1* (*TB1*) [12,13].

The rice HD-ZIP I subfamily gene *OsHOX4* regulates plant growth by participating in the biosynthesis and metabolism pathways of gibberellic acid (GA). Overexpression of *OsHOX4* resulted in dwarfism and an increase in tiller number [14]. The HD-ZIP II subfamily gene *OsHOX3* regulates GA biosynthesis, influencing the growth and development of multiple tissues and organs throughout the entire rice lifecycle. Mutant plants showed fewer tillers and primary branches, shorter panicles and internodes, and reduced grain length, grain width, and 1000-grain weight [15].

Leaves are the main sites for photosynthesis, respiration, and transpiration in plants. Features such as leaf size, shape, thickness, and leaf angle affect light energy utilization, which in turn influences crop yield [16]. Therefore, leaf morphology is one of the key targets in plant architecture improvement. Studies had reported that HD-ZIP III and HD-ZIP IV subfamilies genes participate in regulating leaf morphology development. The rice HD-ZIP III subfamily consists of five genes. Among them, *OsHB1*/*HOX10*, *OsHB2*/*HOX9*, *OsHB3*/*HOX33*, and *OsHB4*/*HOX32* are expressed in specific regions of the shoot apical meristem (SAM), the near-axial cells of the leaf primordium, the leaf margin, and the vascular bundles of the xylem, while *OsHB5*/*HOX29* is expressed only in the phloem tissue [17]. The HD-ZIP III transcription factor *OsHB1*/*HOX10*, also known as *Lateral Floret 1* (*LF1*), regulates auxin distribution by directly controlling the expression of *OsYUCCA6*, maintaining the adaxial–abaxial tissue development balance in rice leaves. Overexpression of *OsHB4*/*HOX32* resulted in various plant phenotypes, including narrow leaves that roll towards the adaxial side, reduced leaf angles, an erect plant phenotype, dwarfism, and decreased photosynthetic rate [18]. OsROC5 and OsROC8 are transcription factors of the HD-ZIP IV subfamily. These two transcription factors, along with the transcriptional repressor cofactor TPL2, form a transcriptional repression complex that directly binds to the promoter of the positively regulated factor *Abaxially Curled Leaf 1* (*OsACL1*) and suppresses its expression, thereby regulating leaf development [19]. In the absence of the *OsROC5* and *OsROC8* genes, the number and size of bulliform cells on the abaxial side of the leaf increased, causing leaf rolling, whereas overexpression of the *OsROC5* and *OsROC8* genes resulted in a reduction in both the number and the size of bulliform cells, leading to adaxially rolled leaves and an upright plant architecture. There were no differences in plant height, panicle shape, fertility, or grain size compared to wild-type (WT) plants [20,21]. Therefore, utilizing the *OsROC5* and *OsROC8* genes for crop improvement to adapt cultures to high-density planting conditions has potential application prospects.

Inflorescence is an important factor that affects crop yield [22]. The rice HD-ZIP III subfamily transcription factor *OsHB1/HOX10* is not only involved in the development of lateral floral meristems but also directly activates the expression of the rice meristem-maintaining gene *OSH1*, triggering the initiation of the inflorescence meristem and participating in the development of spikelets [23]. In barley, the panicle type, whether bi-rowed or six-rowed, is primarily controlled by a single gene, HD-ZIP I type *Vrs1* (*HvHox1*). A mutation in this gene causes a transition from a bi-rowed to a six-rowed spike type [24]. Research has shown that a single amino acid substitution at a phosphorylation site of the barley *Vrs1* gene inhibits the development of lateral flowers, reduces plant height, increases grain size, significantly increases 1000-grain weight, and enhances yield [25]. The rice HD-ZIP I genes *OsHOX12* and *OsHOX14* are homologues of *Vrs1* and are specifically highly expressed in the inflorescence tissues. The OsHOX12 and OsHOX14 transcription factors bind to the promoter of *Elongated Uppermost Internode 1* (*EUI1*) and activate its expression, leading to dwarfism and the inhibition of inflorescence elongation in rice [26,27]. Overexpression of these two genes results in semi-dwarf plants with defective elongation of the uppermost internode, causing the spike to close and obstruct normal pollination, which significantly reduces rice yield [27]. Floret fertility is a key indicator determining the number of grains per spike, which directly impacts wheat yield. The wheat HD-ZIP I transcription factor *Grain Number Increase 1* (*TaGNI1*) is homologous to *Vrs1*. It is specifically expressed in the most apical floret primordia and in parts of the rachilla, suppresses the development of the spikelet axis, and reduces the number of grains per spike [28]. During wheat evolution and selection, the expression level of *GNI1* gradually decreased, leading to an increase in the number of fertile small flowers and grains per spike. Mutation or silencing of this gene significantly increases the fertility rate of small flowers [28].

The tissue-specific expression of genes may have specific biological functions in organisms, with embryonic or seed-specific expressed genes playing important roles in plant reproduction. The maize HD-ZIP IV subfamily gene *Outer Cell Layer 4* (*ZmOCL4*) is specifically expressed in immature vegetative and reproductive organs, such as the margin of young leaf primordia and the inner walls of the anther locules. This suggests that the expression of the *ZmOCL4* gene may lay the foundation for the development and maturation of these organs. Mutation of the *ZmOCL4* gene suppresses trichome development and affects the division and differentiation of the anther cell wall [29].

The maize HD-ZIP IV subfamily gene *Outer Cell Layer 1* (*ZmOCL1*) is specifically expressed in the embryonic outer cell layer and plays a crucial role in early grain development [30,31]. Research has found that *ZmOCL1* regulates the expression of several downstream genes related to lipid transport and metabolism, participating in the biosynthesis and deposition of the plant cell wall cuticle. Overexpression of the *ZmOCL1* gene alters the cuticular wax compounds in maize [32] and delays the flowering time by downregulating the expression of *Zea mays MADS-box 4* (*ZMM4*) and upregulating the expression of *delayed flowering 1* (*DLF1*) [33].

In recent years, with the rapid development of bioinformatics, various molecular biology databases have provided a wealth of nucleotide and amino acid sequences. By using bioinformatics methods to analyze and compute these existing nucleotide and amino acid sequence databases and searching for unique structural features of target sequences (such as conserved domains, functional modification sites, subcellular localization signals, etc.), preliminary function predictions of the target gene have become the fastest and most effective method for discovering new genes and predicting protein structures.

This study used bioinformatics tools to predict and analyze the family members, gene structure, cis-acting elements, and expression patterns of ZmHD-ZIP transcription factors. Many *ZmHD-ZIP* genes were expressed in seeds through expression pattern analysis, and it was found that *ZmHD*-*ZIP23* positively regulates seed size. The results of this study lay the foundation for further research on the function of ZmHD-ZIP transcription factors.

## 2. Results

### 2.1. Identification of ZmHD-ZIP Family Members and Physicochemical Property Analysis

To identify the members of the HD-ZIP family in maize, we searched the Pfam, plantPFDB, and Uniprot databases. Based on the HB, LZ, and START domains, we performed sequence alignment and removed redundant or atypical domain proteins. A total of 61 ZmHD-ZIP proteins were identified. These genes were sequentially named from *ZmHD-ZIP01* to *ZmHD-ZIP61* according to their chromosomal positions (Table 1).

Physicochemical property analysis of the ZmHD-ZIP proteins revealed that they contain from 187 to 863 amino acids (aa), with an average of 483 aa. The molecular weight (MW) of the proteins ranges from 20,556.37 to 93,167.97 Da. Proteins in subfamilies I and II are smaller, while those in subfamilies III and IV are larger. Among these proteins, 18 have an isoelectric point (pI) higher than 7, which classifies them as basic proteins, while the others are acidic proteins. The instability index ranges from 42.4 to 74.68, with values greater than 40 indicating unstable proteins. The hydrophobicity index ranges from −0.853 to 0.019, with values below 0 indicating hydrophilic proteins. Excluding ZmHDZ49 and ZmHDZ60, which have hydrophobicity indices greater than 0, all other proteins are hydrophilic. Subcellular localization prediction showed that these proteins are mainly localized in the nucleus (Table 1). These results indicate that the ZmHD-ZIP family proteins exhibit similarities in terms of isoelectric point, hydrophilicity, instability index, and subcellular localization, suggesting that their sequences are conserved.

### 2.2. Analysis of Conserved Motifs, Domains, and Gene Structure of ZmHD-ZIP Family Members

**Figure 1:**
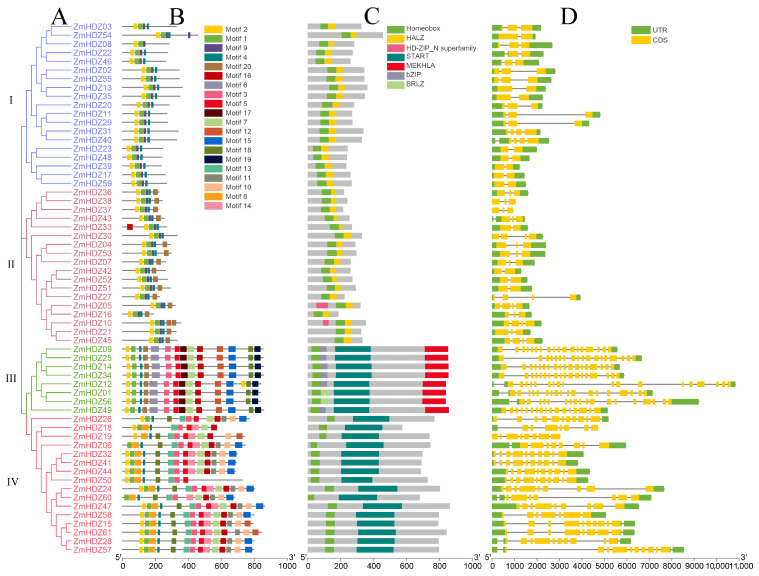
ZmHD-ZIP family gene and protein structure. (**A**) Phylogenetic tree; (**B**) motif analysis; (**C**) conserved domain analysis; (**D**) gene structure.

To elucidate the relationship between the classification and the structure of ZmHD-ZIP family members we analyzed their gene structure, protein domains, and conserved motifs. We constructed a phylogenetic tree for the 61 ZmHD-ZIPs proteins, which were divided into four branches based on their evolutionary relationships (Figure 1A).

Using the MEME tool, we performed a conserved motif analysis on the ZmHD-ZIP family proteins and identified 20 motifs. Visualization analysis was conducted using TBtools v2.225. The results showed that although the number of introns in different genes varies, the proteins within the same subfamily exhibit similar numbers, types, and sequences of motifs, suggesting functional similarity among members of the same subfamily. All four ZmHD-ZIP subfamilies contain motifs 1, 2, and 4, which together form the basic structure of HD-ZIP members. Subfamilies I and II both contain motif 9. Subfamily II (except for ZmHD-ZIP30 and ZmHD-ZIP33) and subfamily III contain motif 20, with motif 20 located near the C-terminus in subfamily II and near the N-terminus in subfamily III. Subfamilies III and IV do not contain motif 9, but both contain motifs 3, 5, 7, 15, 16, and 18. Notably, motif 18 in subfamily III is located near the C-terminus, while in subfamily IV, it is located near the N-terminus. The unique conserved motifs in subfamily III are motifs 6, 12, 17, and 19, while subfamily IV has unique conserved motifs 8, 10, 11, 13, and 14. The specific motifs in each subfamily are associated with their respective biological functions (Figure 1B).

Domain analysis revealed that all ZmHD-ZIP family members contain a homeobox domain. Subfamilies I and II both have a HALZ domain, while subfamilies III and IV contain a START domain. Subfamily III uniquely contains a MEKHLA domain. Furthermore, the distribution of the domains aligns perfectly with the subfamily classification criteria, with members of the same subfamily sharing similar domains (Figure 1C).

Gene structure analysis revealed differences among ZmHD-ZIP members. The coding regions of the genes in subfamilies I and II contain 1 to 3 introns, making their structure simpler compared to that of the genes in subfamilies III and IV. In subfamily III, except for *ZmHDZ49*, which has 16 introns, the remaining members have 17 introns. In subfamily IV, the number of introns ranges from 6 to 10. These results indicate that genes within the same subfamily have similar intron and exon structures, while there are significant differences in the intron and exon structures between different subfamilies (Figure 1D).

### 2.3. Phylogenetic Analysis of ZmHD-ZIP Family Members

**Figure 2:**
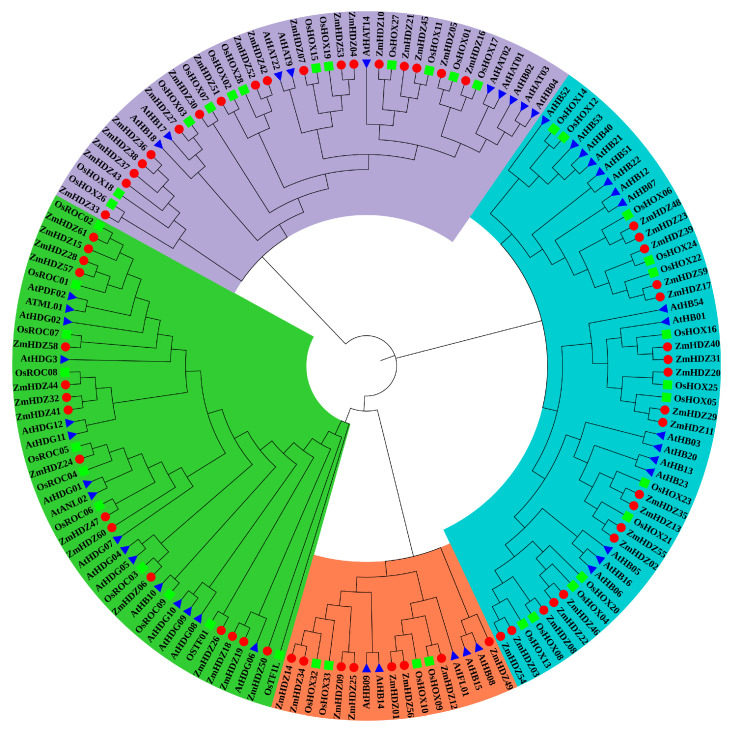
Analysis of the evolutionary relationship of HD-ZIP genes in maize, rice, and Arabidopsis. Blue represents the HD-ZIP I subfamily; purple represents the HD-ZIP II subfamily; red represents the HD-ZIP III subfamily; green represents the HD-ZIP IV subfamily. The red circles represent HD-ZIP members in maize; the blue triangles represent HD-ZIP members in Arabidopsis; the green squares represent HD-ZIP members in rice.

To better understand the evolutionary relationships of HD-ZIP family members, we constructed a phylogenetic tree for maize, rice, and Arabidopsis (Figure 2). The results showed that the ZmHD-ZIP family consists of four subfamilies: HD-ZIP I, HD-ZIP II, HD-ZIP III, and HD-ZIP IV. Among these, ZmHD-ZIP I has the largest number of members, accounting for 31.15% of the total family members, while ZmHD-ZIP III has the fewest members, accounting for 13.11% of the total number. The phylogenetic tree showed that ZmHD-ZIP III and ZmHD-ZIP IV are located in the same branch, suggesting that their evolutionary relationship may be close (Figure 2).

### 2.4. Cis-Acting Element Analysis of ZmHD-ZIP Genes

**Figure 3:**
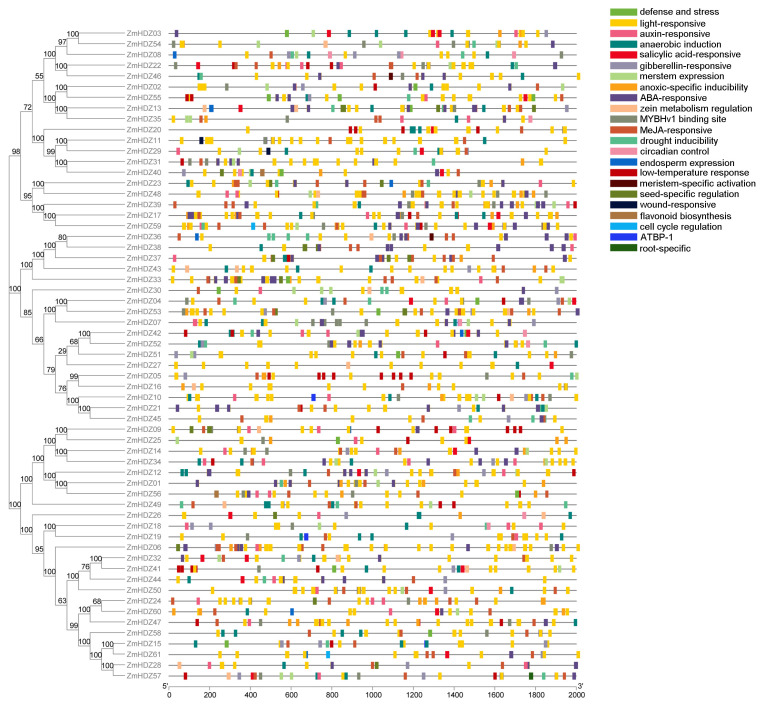
Analysis of cis-acting regulatory elements in *ZmHD-ZIP* genes. The key cis-acting regulatory elements are distributed in the 2000 bp region upstream of the *ZmHD-ZIP* genes, and different elements are shown in different colors.

To explore the functions of the genes in this family, we conducted a cis-acting element analysis of the ZmHD-ZIP family. The results showed that, in addition to core enhancer elements, several hormone-related elements were identified, including GA, auxin (IAA), abscisic acid (ABA), and methyl jasmonate (MeJA) response elements, as well as cis-acting elements related to abiotic stress, such as low-temperature response elements (LTR). Moreover, seed-specific regulatory elements and endosperm regulatory elements were also found (Figure 3). These results suggest that the ZmHD-ZIP family genes are involved not only in abiotic stress regulation but also, potentially, in the regulation of plant reproductive development.

### 2.5. Chromosome Localization and Collinearity Analysis of ZmHD-ZIP Genes

**Figure 4:**
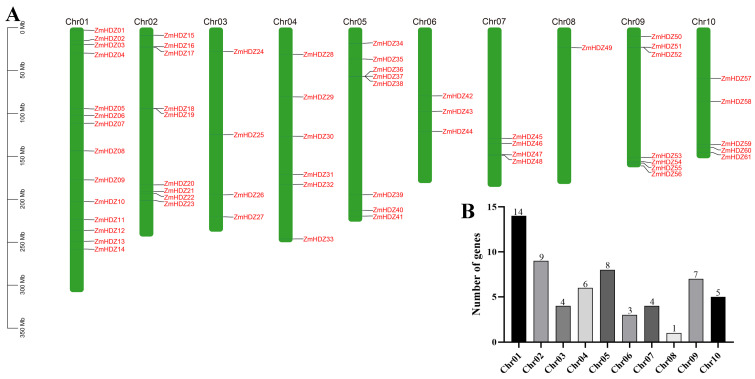
The location of the *ZmHD-ZIP* genes on chromosomes. (**A**) Distribution of the *ZmHD-ZIP* genes on chromosomes; (**B**) the number of *ZmHD-ZIP* genes on each chromosome.

To understand the distribution of the *ZmHD-ZIP* genes on the chromosomes, we performed a chromosome localization analysis of the family members. The results showed that the 61 *ZmHD-ZIP* genes are unevenly distributed across 10 chromosomes (Figure 4). The largest number of genes are located on chromosome 1, with 14 genes, accounting for 23% of the total, while the fewest genes are located on chromosome 8, with only 1 gene, accounting for 1.6% of the total. The number of genes on the remaining chromosomes ranges from 3 to 9, with genes on chromosomes 5 and 9 located at the ends of the chromosomes, while the remaining *ZmHD-ZIP* genes are randomly distributed along the chromosomes.

**Figure 5:**
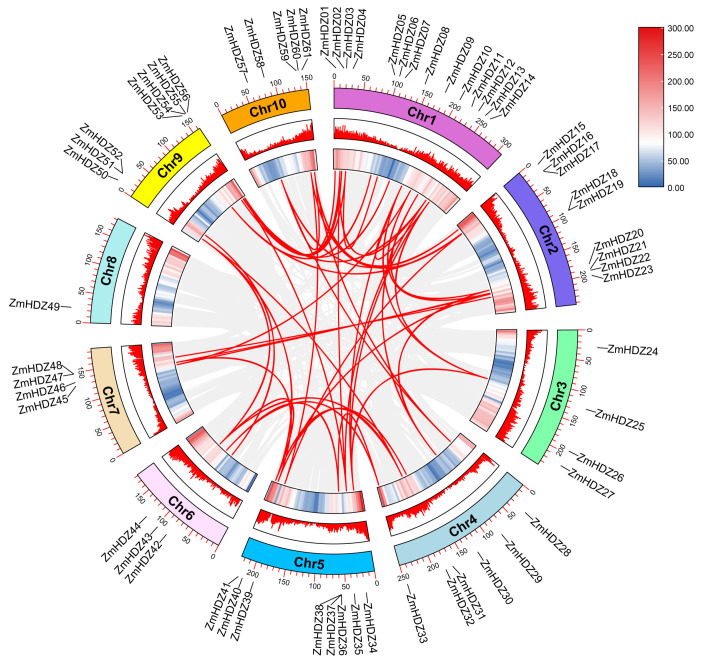
Collinearity analysis of the ZmHD-ZIP genes. Heat maps and histograms along the rectangles represent gene densities on chromosomes. Gray lines indicate syntenic blocks in the maize genome, and red lines between chromosomes indicate gene pairs with segmental duplications.

Tandem duplication and segmental replication are the main driving forces for gene family expansion, promoting the formation of gene families and genome evolution. Using TBtools v2.225 and MCScanX for tandem repeat analysis, no repeat events were observed. However, fragment repeat analysis revealed 48 gene pairs, with 20 pairs located on chromosome 1 (Figure 5). These results suggest that fragment repeat events are a major driving force behind the diversity of the *ZmHD-ZIP* genes. We further analyzed the Ka, Ks, and Ka/Ks values of ZmHD-ZIP replicated gene pairs (Table S1). The results showed that the Ka/Ks values of ZmHD-ZIP replicated gene pairs were all less than 1.0 (0.118–0.530), indicating that ZmHD-ZIP gene pairs underwent purification selection during evolution and suggesting that the function of the ZmHD-ZIP genes is relatively conserved.

**Figure 6:**
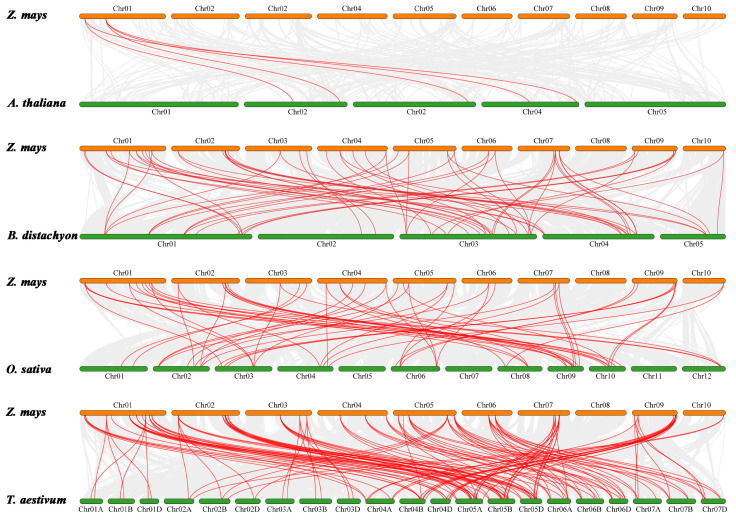
Collinearity analysis of HD-ZIP genes in maize, Arabidopsis, wheat, and *Brachypodium distachyon* L. Red lines between chromosomes indicate gene pairs with segmental duplications.

Additionally, we explored the collinearity relationships of *ZmHD-ZIP* genes in four representative species, including the dicotyledonous Arabidopsis and three monocots—rice, wheat, and *Brachypodium distachyon* L.—to identify orthologous genes. The results showed 5 pairs of orthologous genes between maize and Arabidopsis, 60 pairs between maize and *Brachypodium distachyon* L., 55 pairs between maize and rice, and 124 pairs between maize and wheat (Figure 6). These results suggest that maize has a closer phylogenetic relationship with wheat.

### 2.6. Expression Pattern Analysis of ZmHD-ZIP Family Members

**Figure 7:**
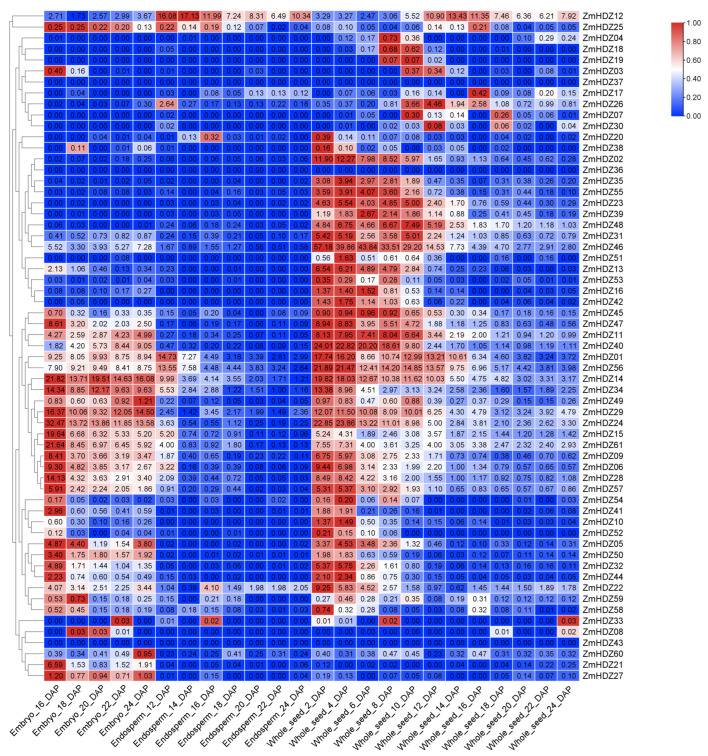
*ZmHD-ZIP* genes expressed in different seed parts in different periods. The transverse axis represents different seed parts in different periods, and the longitudinal axis represents the *ZmHD-ZIP* genes.

To predict the expression patterns of *ZmHD-ZIP* genes in seeds, we constructed a heatmap of *ZmHD-ZIP* expression in different parts of the seed. The results showed that most genes in this family are expressed in various parts of the seed. Among them, genes such as *ZmHD-ZIP02*, *ZmHD-ZIP13*, *ZmHD-ZIP16*, *ZmHD-ZIP23*, *ZmHD-ZIP35*, *ZmHD-ZIP42*, *ZmHD-ZIP53*, and *ZmHD-ZIP55* exhibited low expression levels in the embryo and endosperm, with their expression levels increasing initially in the seed, reaching a peak, and then decreasing. Genes like *ZmHD-ZIP05*, *ZmHD-ZIP14*, *ZmHD-ZIP29*, *ZmHD-ZIP49*, *ZmHD-ZIP58*, *ZmHD-ZIP59*, and *ZmHD-ZIP60* are expressed in both embryo and seed. However, genes such as *ZmHD-ZIP36*, *ZmHD-ZIP37*, and *ZmHD-ZIP43* are not expressed at any time or in any spatial location during seed development (Figure 7). These findings suggest that most genes in this family may regulate seed development.

### 2.7. ZmHD-ZIP23 Positively Regulates Seed Size

**Figure 8:**
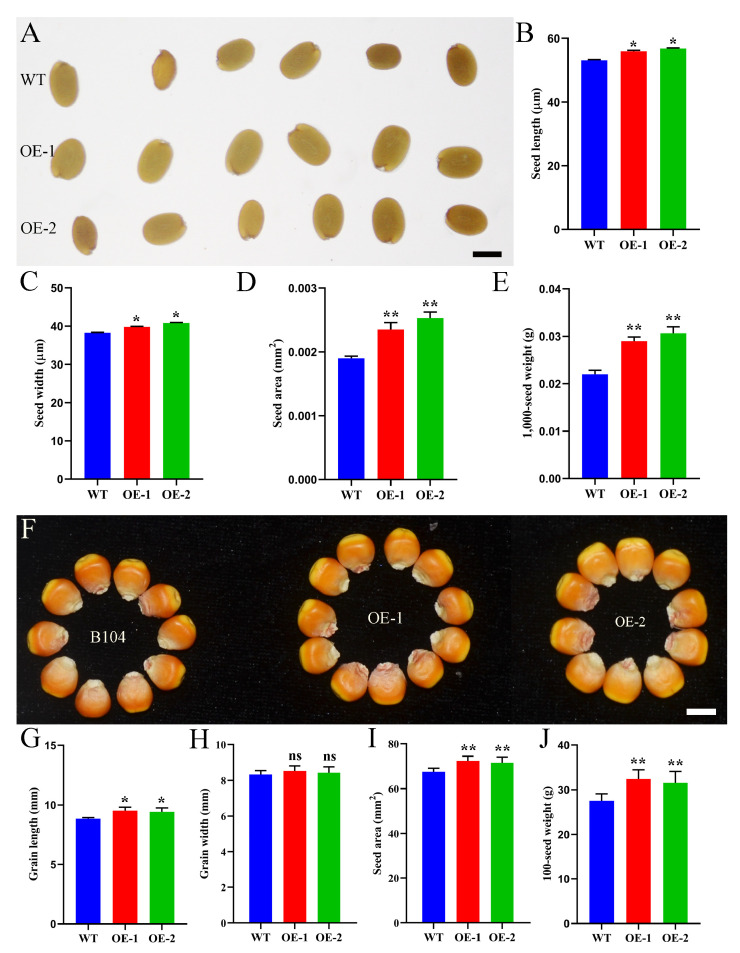
ZmHD-ZIP23 positively regulates seed size. (**A**) Seeds of WT line, *ZmHD-ZIP23*-overexpressing line 1 (OE-1), and *ZmHD-ZIP23*-overexpressing line 2 (OE-2) of Arabidopsis. (**B**–**E**). Statistical data on seed length (**B**), seed width (**C**), seed area (**D**), and 1000-seed weight (**E**) of WT, OE-1, and OE-2 Arabidopsis plants. (**F**) Grains of B104 line, *ZmHD-ZIP23*-overexpressing line 1 (OE-1), and *ZmHD*-*ZIP23*-overexpressing line 2 (OE-2) of maize. (**G**–**J**) Statistical data for grain length (**G**), grain width (**H**), seed area (**I**), and 100-seed weight (**J**) for B104, OE-1, and OE-2 maize plants. The expression level of *ZmHD-ZIP23* in OE-1 and OE-2 was higher than the WT (Figure S1).The data represent the mean ± SD of three biological repeats, and the asterisks represent significant differences among groups (* *p* < 0.05, ** *p* < 0.01); ns, no significant differences. Bars, 40 µm (**A**), 0.5 cm (**F**).

To explore the function of *ZmHD-ZIP* family members in seed development, we constructed transgenic Arabidopsis plants with overexpression of *ZmHD-ZIP02*, *ZmHD-ZIP05*, *ZmHD-ZIP13*, *ZmHD-ZIP14*, *ZmHD-ZIP16*, *ZmHD-ZIP23*, *ZmHD-ZIP29*, *ZmHD-ZIP35*, *ZmHD-ZIP42*, *ZmHD-ZIP49*, *ZmHD-ZIP53*, *ZmHD-ZIP55*, *ZmHD-ZIP58*, *ZmHD-ZIP59*, and *ZmHD-ZIP60*. Homozygous lines were obtained through self-crossing for four generations. The results showed that the seeds of the *ZmHD-ZIP23*-overexpressing lines were larger than those of the WT line (Figure 8A), with greater length (Figure 8B) and width (Figure 8C), bigger seed area (Figure 8D), and higher 1000-seed weight (Figure 8E), indicating that *ZmHD-ZIP23* positively regulates seed size in Arabidopsis.

To further explore the role of *ZmHD-ZIP23* in maize grain development, we constructed *ZmHD*-*ZIP23*-overexpressing maize plants. To compare the yield of field-harvested B104 maize with that of *ZmHD*-*ZIP23*-overexpressing maize plants, we measured and recorded seed area, grain length, grain width, and 100-grain weight. The results showed that *ZmHD*-*ZIP23*-overexpressing maize plants had larger grains (Figure 8F), significantly increased grain length (Figure 8G), no significant change in grain width (Figure 8H), larger seed area (Figure 8I), and heavier 100-grain weight (Figure 8J). These results indicate that the grains of *ZmHD*-*ZIP23*-overexpressing maize plants were superior to those of B104 in both size and weight, suggesting that this gene may play an important role in improving maize yield.

### 2.8. ZmHD-ZIP23-Overexpressing Maize Has Bigger Embryo and Endosperm

**Figure 9:**
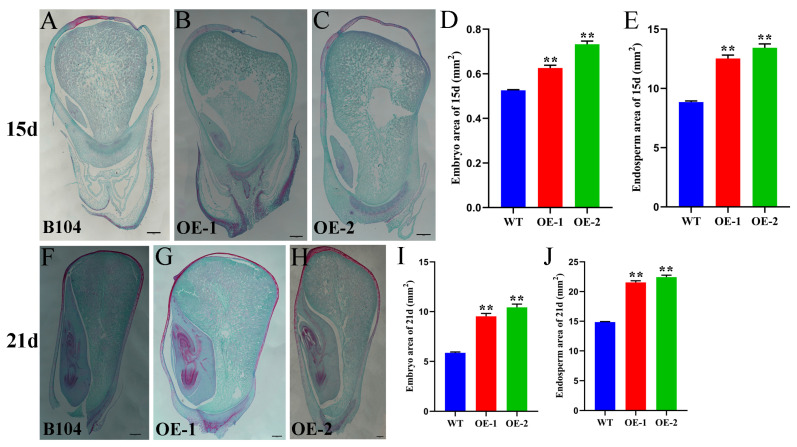
Paraffin sections of ZmHD-ZIP23-overexpressing maize seed. (**A**–**C**) Paraffin sections of 15-day maize seeds from B104 maize (**A**), *ZmHD-ZIP23*-overexpressing line 1 (OE-1) (**B**), *ZmHD*-*ZIP23*-overexpressing line 2 (OE-2) (**C**). (**D**,**E**) Statistical data for embryo area (**D**) and endosperm area (**E**) for B104, OE-1, and OE-2 lines. (**F**–**H**) Paraffin sections of 21-day maize seeds from B104 (**F**), OE-1 (**G**), OE-2 (**H**) lines. (**I**,**J**). Statistical data for embryo area (**I**) and endosperm area (**J**) for B104, OE-1, and OE-2 lines. The data represent the mean ± SD of three biological repeats, and the asterisks represent significant differences among groups (** *p* < 0.01). Bars, 500 µm.

The seed size depends on the size of the embryo and endosperm. In order to investigate the size of embryo and endosperm during the development of *ZmHD-ZIP23*-overexpressing maize, samples were taken from seeds at 15 and 21 days after pollination, and paraffin sections were prepared. Microscopic observation revealed that the embryo and endosperm areas of OE-1 and OE-2 were significantly larger than those of B104 at both 15 and 21 d (Figure 9). This indicates that during seed development, the seeds of *ZmHD-ZIP23*-overexpressing maize developed faster than those of B104 maize, had larger embryo and endosperm, and might accumulate more nutrients at the same time, which is of great significance for increasing maize seed yield.

## 3. Discussion

### 3.1. Evolutionary and Structural Conservation of the ZmHD-ZIP Family

Corn is an important food and feed crop, with the highest total production worldwide, and has significant economic value. Conducting in-depth research on the functions of maize genes in growth and development processes is of great importance [34,35].

HD-ZIP proteins are plant-specific transcription factors that play an important regulatory role in plant growth and development. Research has shown that genes from the same HD-ZIP subfamily have similar functions across different species, with conserved features [36]. However, due to the complexity of gene structures and differences between species, functional differences may arise. Therefore, further exploration of new HD-ZIP genes, their functions, and the regulatory networks they are involved in requires more extensive and in-depth research.

Our systematic identification and analysis of 61 *ZmHD-ZIP* genes in maize revealed conserved physicochemical properties, including similar isoelectric points, hydrophilicity, and nuclear localization (Table 1), suggesting functional conservation across subfamilies. Phylogenetic analysis classified these proteins into four subfamilies (I–IV), consistent with prior reports in *Arabidopsis* and rice (Figure 1A) [1,2]. Notably, subfamilies III and IV clustered together (Figure 2), implying a closer evolutionary relationship, possibly due to shared domain architectures (e.g., START domain) (Figure 1C). The absence of tandem duplications and the presence of 48 segmentally duplicated pairs (Figure 5) highlights fragment replication as a key driver of ZmHD-ZIP diversification, supported by Ka/Ks values < 1 (purifying selection) (Table S1). These findings align with studies in rice [3], reinforcing the role of purifying selection in maintaining conserved functions for HD-ZIP genes.

### 3.2. Subfamily-Specific Motifs and Functional Implications

Distinct motif compositions among subfamilies (e.g., motif 9 in subfamilies I/II vs. motifs 3–18 in subfamilies III/IV; Figure 1B) correlate with their functional divergence. For instance, the MEKHLA domain, unique to subfamily III (Figure 1C), is associated with stress responses [4], while the HALZ domains in subfamilies I/II have roles in dimerization [5]. Gene structure analysis further supported subfamily divergence, with simpler intron–exon patterns in subfamilies I/II versus complex structures in subfamilies III/IV (Figure 1D). Such structural variations may underlie differential regulatory mechanisms, as observed for *Arabidopsis* HD-ZIP members [6].

### 3.3. Cis-Elements and Expression Patterns Suggest Multifunctionality

HD-ZIP family genes mediate the expression of downstream related genes by integrating endogenous genetic information and exogenous environmental signals, initiating corresponding physiological and biochemical activities to regulate plant growth, development, and responses to stress. As transcription factors, these genes regulate downstream gene expression but may also be regulated by other upstream genes or interact with proteins to exert their effects. Therefore, the multi-level regulation of transcription factor activity, downstream gene expression, and protein interactions forms a complex regulatory network [37,38].

The presence of hormone-responsive (e.g., ABA, MeJA) and abiotic stress-related elements (e.g., LTR; Figure 3) implies ZmHD-ZIP involvement in stress adaptation and development. Seed-specific expression of genes like *ZmHD-ZIP23* (Figure 7) and their dynamic expression profiles during embryogenesis suggest roles in reproductive development, corroborating findings in wheat [7]. Notably, the lack of expression of *ZmHD-ZIP36/37/43* may indicate pseudogenization or context-specific regulation.

### 3.4. ZmHD-ZIP23 as a Key Regulator of Seed Size and Yield

Overexpression of *ZmHD-ZIP23* significantly increased seed size and weight in both *Arabidopsis* and maize (Figure 8A–J), likely through promoting embryo and endosperm expansion (Figure 9). This phenotype mirrors *OsHD-ZIP24*-mediated grain enlargement in rice [8], suggesting a conserved role for HD-ZIP III members in seed development. The absence of grain width changes in maize (Figure 8H) contrasts with what observed in *Arabidopsis*, possibly due to species-specific regulatory networks. Enhanced nutrient accumulation in *ZmHD-ZIP23*-OE endosperm (Figure 9) further underscores its agronomic potential for yield improvement.

### 3.5. Limitations and Prospects

While our study provides comprehensive genomic and functional insights, the exact mechanisms by which *ZmHD-ZIP23* regulates seed size remain unclear. How HD-ZIP participates in signal transduction pathways and responds to endogenous upstream signals or exogenous stimuli, what the upstream regulators are, how they regulate the expression of downstream functional genes, how interacting proteins affect the activity of these transcription factors, how to use gene editing such as CRISPR/Cas [39] to improve crops—all of these questions require continuous exploration by researchers. Additionally, field trials across multiple environments are needed to validate yield stability.

## 4. Materials and Methods

### 4.1. Identification and Analysis of the Physiochemical Properties of the ZmHD-ZIP Family Members

To identify the members of the ZmHD-ZIP family, genome annotation files were downloaded from EnsemblPlants (https://plants.ensembl.org/index.html, accessed on 1 May 2025). Using the ZmHD-ZIP protein sequences, the family was screened, and its members were identified with TBtools v2.225 [40,41] software combined with BLAST (https://blast.ncbi.nlm.nih.gov/Blast.cgi, accessed on 1 May 2025). A total of 61 ZmHD-ZIP proteins were identified. The physiochemical properties of these ZmHD-ZIP proteins were predicted using ExPASy (https://www.expasy.org, accessed on 1 May 2025) [42], and their subcellular localization was predicted using Plant-mPLoc 2.0 tool (http://www.csbio.sjtu.edu.cn/bioinf/plant-multi/, accessed on 1 May 2025) [43].

### 4.2. Analysis of Conserved Motifs, Domains, and Gene Structure

Gene Structure Display Server 2.0 (GSDS 2.0) (http://gsds.gao-lab.org/, accessed on 2 May 2025) [44,45] was used to construct the gene structure map. The conserved motifs in the ZmHD-ZIP family genes were predicted by MEME (https://meme-suite.org/meme/tools/meme, accessed on 2 May 2025) [46,47].

### 4.3. Phylogenetic Analysis of ZmHD-ZIP Genes

Multiple sequence alignment was performed using MEGA X [48] software with the neighbor-joining (NJ) method. A phylogenetic tree was then constructed based on the HD-ZIP protein sequences from maize, rice, and Arabidopsis. The resulting file was imported into iTOL v6 (http://itol.embl.de/, accessed on 3 May 2025) [49] for visualization.

### 4.4. Cis-Acting Element Analysis of ZmHD-ZIP Genes

The promoter regions 2000 bp upstream of the *ZmHD-ZIP* genes were extracted using TBtools v2.225, and the cis-acting elements were predicted using PlantCARE (http://bioinformatics.psb.ugent.be/webtools/plantcare/html/, accessed on 3 May 2025) [50]. The results were visualized using TBtools v2.225.

### 4.5. Chromosomal Localization and Synteny Analysis of ZmHD-ZIP Genes

Using the maize genome annotation file, the chromosomal localization of *ZmHD-ZIP* genes was performed using TBtools v2.225, and the result files were imported into TBtools v2.225 for visualization. MCScanX tool (https://github.com/wyp1125/MCScanX, accessed on 4 May 2025) [51] was used for synteny analysis of the ZmHD-ZIP genes, followed by visualization with TBtools v2.225.

### 4.6. Expression Pattern Analysis of ZmHD-ZIP Genes

The specific expression data of the *ZmHD-ZIP* genes were downloaded from MaizeGDB-qteller (https://qteller.maizegdb.org, accessed on 4 May 2025), and a heatmap was generated using TBtools v2.225.

### 4.7. Vector Construction

The CDS sequences of *ZmHD-ZIP02*, *ZmHD-ZIP05*, *ZmHD-ZIP13*, *ZmHD-ZIP14*, *ZmHD-ZIP16*, *ZmHD-ZIP23*, *ZmHD-ZIP29*, *ZmHD-ZIP35*, *ZmHD-ZIP42*, *ZmHD-ZIP49*, *ZmHD-ZIP53*, *ZmHD-ZIP55*, *ZmHD-ZIP58*, *ZmHD-ZIP59*, and *ZmHD-ZIP60* were recombined into the plant binary expression vector pcambimGFP-1302 using ClonExpress technology (https://www.vazyme.com, accessed on 27 May 2023). The primers for gene cloning are listed in Table S2.

### 4.8. Generation of Transgenic Arabidopsis

The genetic transformation of *Arabidopsis thaliana* was based on the *Agrobacterium*-mediated floral dip method [52]. After T_1_ transgenic plants were obtained, we identified the positive plants by PCR using a 35S sequence primer as the forward primer and a gene-specific primer as the reverse primer (Table S3). A minimum of 12 positive transgenic lines were identified [53].

### 4.9. Generation of Transgenic Maize

The CDS sequence of *ZmHD-ZIP23* was cloned and recombined into a modified pCambia3300 vector. ZmUbiquitin1 (ZmUbi) was used as the promoter to drive *ZmHD-ZIP23* expression. The *ZmHD-ZIP23* overexpression construct was transformed into the maize inbred line B104 provided by the Henan Academy of Agricultural Sciences, Zhengzhou, China. A minimum of 6 positive transgenic lines were identified.

## 5. Conclusions

In this study, we conducted a phylogenetic analysis of ZmHD-ZIP family members using bioinformatics methods. The results revealed that 61 ZmHD-ZIPs were identified in maize, and the family was divided into four subfamilies: HD-ZIP I, HD-ZIP II, HD-ZIP III, and HD-ZIP IV. Physicochemical property analysis showed that the ZmHD-ZIP family proteins exhibit similarities in terms of isoelectric point, hydrophilicity, instability index, and subcellular localization, suggesting that their sequences are conserved. Domain analysis revealed that the distribution of the domains aligns perfectly with the subfamily classification criteria, with members of the same subfamily sharing similar domains. Cis-acting element analysis suggested that the ZmHD-ZIP family genes are involved in the regulation of plant reproductive development. Expression pattern analysis of the ZmHD-ZIP family genes suggested that most genes in this family may regulate seed development. In addition, *ZmHD-ZIP23*-overexpressing Arabidopsis and maize had larger seeds with increased grain length and heavier grain weight owing to bigger embryo and endosperm area. Our integrated genomic and phenotypic analyses bridge evolutionary biology and crop biotechnology, offering a framework for future functional studies.

## Figures and Tables

**Table 1:** Physicochemical properties of ZmHD-ZIP proteins identified in maize.

Genes	Gene ID	Amino Acids	MW (Da)	pI	Instability Index	GRAVY	Subcellular Localization
*ZmHD-ZIP01*	Zm00001eb000690	840	92,098.18	5.84	53.84	−0.125	Nucleus
*ZmHD-ZIP02*	Zm00001eb005440	344	37,604.1	6.28	58.58	−0.696	Nucleus
*ZmHD-ZIP03*	Zm00001eb006700	326	35,531.37	5.11	62.69	−0.739	Nucleus
*ZmHD-ZIP04*	Zm00001eb009550	290	30,707.68	8.69	54.27	−0.492	Nucleus
*ZmHD-ZIP05*	Zm00001eb023620	321	34,828.19	8.86	63.2	−0.69	Nucleus
*ZmHD-ZIP06*	Zm00001eb024680	746	80,347.87	5.95	48.17	−0.188	Nucleus
*ZmHD-ZIP07*	Zm00001eb025650	262	28,241.88	8.43	48.86	−0.508	Nucleus
*ZmHD-ZIP08*	Zm00001eb027860	283	30,941.65	4.75	59.27	−0.71	Nucleus
*ZmHD-ZIP09*	Zm00001eb031670	854	92,572.62	6.54	49.04	−0.125	Nucleus
*ZmHD-ZIP10*	Zm00001eb037670	354	36,671.05	8.16	56.32	−0.369	Nucleus
*ZmHD-ZIP11*	Zm00001eb042400	270	29,994.23	5	56.95	−0.665	Nucleus
*ZmHD-ZIP12*	Zm00001eb045630	841	92,210.07	5.83	48.9	−0.17	Nucleus
*ZmHD-ZIP13*	Zm00001eb048440	363	38,897.15	5.74	70.68	−0.666	Nucleus
*ZmHD-ZIP14*	Zm00001eb050660	856	93,167.97	6.16	47.74	−0.188	Nucleus
*ZmHD-ZIP15*	Zm00001eb070050	794	85,194.04	5.52	42.77	−0.25	Nucleus
*ZmHD-ZIP16*	Zm00001eb074830	187	20,556.37	9.06	61.44	−0.639	Nucleus
*ZmHD-ZIP17*	Zm00001eb075230	261	29,384.76	5	71.3	−0.715	Nucleus
*ZmHD-ZIP18*	Zm00001eb087220	574	64,224.37	5.46	48	−0.279	Nucleus
*ZmHD-ZIP19*	Zm00001eb087250	741	80,705.57	5.04	47.82	−0.265	Nucleus
*ZmHD-ZIP20*	Zm00001eb098960	283	31,224.78	5.34	67.03	−0.644	Nucleus
*ZmHD-ZIP21*	Zm00001eb100690	325	34,517.69	7.6	58.03	−0.515	Nucleus
*ZmHD-ZIP22*	Zm00001eb101280	274	29,643.92	4.61	61.83	−0.486	Nucleus
*Zm* *HD-ZIP23*	Zm00001eb103330	244	26,792.74	5.36	57.74	−0.786	Nucleus
*ZmHD-ZIP24*	Zm00001eb126140	803	85,946.16	5.44	51.43	−0.145	Nucleus
*ZmHD-ZIP25*	Zm00001eb136060	854	92,757.92	6.37	49.21	−0.142	Nucleus
*ZmHD-ZIP26*	Zm00001eb151130	770	83,567.83	6.14	45.76	−0.313	Nucleus
*ZmHD-ZIP27*	Zm00001eb158680	225	24,891.12	9.27	66.93	−0.837	Nucleus
*ZmHD-ZIP28*	Zm00001eb171720	796	85,608.48	5.53	46	−0.259	Nucleus
*ZmHD-ZIP29*	Zm00001eb179000	272	30,035.08	4.79	54.13	−0.679	Nucleus
*ZmHD-ZIP30*	Zm00001eb182650	331	35,536.87	5.49	70.03	−0.438	Nucleus
*ZmHD-ZIP31*	Zm00001eb189900	339	36,998.47	4.62	53.68	−0.723	Nucleus
*ZmHD-ZIP32*	Zm00001eb193330	698	76,174.03	6.19	53.03	−0.164	Nucleus
*ZmHD-ZIP33*	Zm00001eb208000	269	28,268.55	9.65	61.44	−0.516	Nucleus
*ZmHD-ZIP34*	Zm00001eb218730	856	93,155.96	6.16	47.24	−0.187	Nucleus
*ZmHD-ZIP35*	Zm00001eb223300	348	37,530.79	6.01	65.65	−0.689	Nucleus
*ZmHD-ZIP36*	Zm00001eb226570	221	23,801.92	9.2	54.34	−0.533	Nucleus
*ZmHD-ZIP37*	Zm00001eb226580	216	23,703.89	9.72	68.41	−0.643	Nucleus
*ZmHD-ZIP38*	Zm00001eb226590	241	25,859.12	9.48	61.57	−0.606	Nucleus
*ZmHD-ZIP39*	Zm00001eb248930	235	26,413.07	5.01	56.92	−0.814	Nucleus
*ZmHD-ZIP40*	Zm00001eb253470	330	35,891.27	4.58	56.81	−0.636	Nucleus
*ZmHD-ZIP41*	Zm00001eb256420	692	75,813.55	6.15	48.09	−0.205	Nucleus
*ZmHD-ZIP42*	Zm00001eb270270	261	27,367.76	9.5	64.05	−0.419	Nucleus
*ZmHD-ZIP43*	Zm00001eb273120	254	27,552.84	9.11	71.41	−0.651	Nucleus
*ZmHD-ZIP44*	Zm00001eb278870	687	74,909.39	5.77	49.66	−0.166	Nucleus
*ZmHD-ZIP45*	Zm00001eb314500	333	36,021.26	6.98	74.68	−0.656	Nucleus
*ZmHD-ZIP46*	Zm00001eb315750	261	28,461.52	4.76	57.29	−0.703	Nucleus
*ZmHD-ZIP47*	Zm00001eb319090	863	91,541.38	5.88	47.53	−0.246	Nucleus
*ZmHD-ZIP48*	Zm00001eb319390	239	26,235.01	5.37	46.67	−0.853	Nucleus
*ZmHD-ZIP49*	Zm00001eb337970	858	92,028.36	5.72	45.27	0.019	Nucleus
*ZmHD-ZIP50*	Zm00001eb373520	729	81,395.84	10.54	67.18	−0.725	Nucleus
*ZmHD-ZIP51*	Zm00001eb377480	293	31,098.95	9.37	48.67	−0.532	Nucleus
*ZmHD-ZIP52*	Zm00001eb377500	272	28,026.55	9.45	67.6	−0.318	Nucleus
*ZmHD-ZIP53*	Zm00001eb399360	296	31,560.54	9.21	61.43	−0.651	Nucleus
*ZmHD-ZIP54*	Zm00001eb401450	458	50,056.7	8.61	58.17	−0.24	Nucleus
*ZmHD-ZIP55*	Zm00001eb402220	344	37,573.13	6.04	59.47	−0.639	Nucleus
*ZmHD-ZIP56*	Zm00001eb404260	839	92,090.13	5.94	54.2	−0.138	Nucleus
*ZmHD-ZIP57*	Zm00001eb413140	798	85,633.3	5.43	47.99	−0.295	Nucleus
*ZmHD-ZIP58*	Zm00001eb416980	797	85,648.79	5.97	44.99	−0.256	Nucleus
*ZmHD-ZIP59*	Zm00001eb427650	268	29,290.62	5.32	67.04	−0.63	Nucleus
*ZmHD-ZIP60*	Zm00001eb428740	681	74,199.73	6.21	42.4	0.003	Nucleus
*ZmHD-ZIP61*	Zm00001eb431350	844	91,063.09	5.68	49.88	−0.209	Nucleus

## Data Availability

The original contributions presented in this study are included in the article/Supplementary Materials. Further inquiries can be directed to the corresponding author(s).

## Supplementary Materials

The following supporting information can be downloaded at: https://www.mdpi.com/article/10.3390/plants14162477/s1, Figure S1:The expression level of ZmHD-ZIP23 in Arabidopsis and maize; Table S1: The ratio of Nonsynonymous substitution (Ka) and Synonymous substitution (Ks) of ZmHD-ZIP replication gene pairs in maize; Table S2: Primer sequences for gene cloning; Table S3:Primer sequences for PCR.
