# Supplementary material for: Genome-Wide Identification of the HD-ZIP Transcription Factor Family in Maize and Functional Analysis of the Role of ZmHD-ZIP23 in Seed Size
Source: Plants (Basel). 2025 Aug 10;14(16):2477. doi: 10.3390/plants14162477 (PMC12389044; doi:10.3390/plants14162477)
Supplement: Supplementary file 1 [file plants-14-02477-s001.zip › plants-3687972-supplementary.pdf]

Table S1. The ratio of Nonsynonymous substitution (Ka) and Synonymous substitution (Ks) of ZmHD-ZIP replication gene pairs in maize

Name_1	Name_2	Ka	Ks	Ka/Ks
ZmHD-ZIP01	ZmHD-ZIP53	0.00609	0.04080	0.14926
ZmHD-ZIP02	ZmHD-ZIP54	0.07527	0.47361	0.15892
ZmHD-ZIP03	ZmHD-ZIP55	0.05237	0.43544	0.12026
ZmHD-ZIP04	ZmHD-ZIP56	0.00001	0.00908	0.00110
ZmHD-ZIP05	ZmHD-ZIP53	0.00227	0.02743	0.08275
ZmHD-ZIP06	ZmHD-ZIP04	0.07788	0.50789	0.15334
ZmHD-ZIP07	ZmHD-ZIP53	0.05372	0.46973	0.11436
ZmHD-ZIP08	ZmHD-ZIP11	0.00227	0.02743	0.08275
ZmHD-ZIP09	ZmHD-ZIP47	0.05171	0.46619	0.11092
ZmHD-ZIP10	ZmHD-ZIP20	0.05505	0.46335	0.11880
ZmHD-ZIP11	ZmHD-ZIP21	0.07788	0.49412	0.15761
ZmHD-ZIP12	ZmHD-ZIP54	0.05372	0.45570	0.11788
ZmHD-ZIP13	ZmHD-ZIP34	0.06020	0.38857	0.15492
ZmHD-ZIP14	ZmHD-ZIP35	0.05620	0.41011	0.13703
ZmHD-ZIP15	ZmHD-ZIP60	0.04878	0.32206	0.15146
ZmHD-ZIP16	ZmHD-ZIP61	0.08339	0.62012	0.13447
ZmHD-ZIP17	ZmHD-ZIP39	0.09841	0.53853	0.18273
ZmHD-ZIP20	ZmHD-ZIP09	0.09164	0.62163	0.14741
ZmHD-ZIP21	ZmHD-ZIP10	0.05583	0.32491	0.17183
ZmHD-ZIP22	ZmHD-ZIP30	0.06662	0.52359	0.12723
ZmHD-ZIP23	ZmHD-ZIP46	0.08329	0.53038	0.15703
ZmHD-ZIP25	ZmHD-ZIP08	0.01254	0.03190	0.39310
ZmHD-ZIP28	ZmHD-ZIP60	0.01382	0.03632	0.38050
ZmHD-ZIP29	ZmHD-ZIP21	0.00865	0.04543	0.19040
ZmHD-ZIP31	ZmHD-ZIP40	0.00665	0.05543	0.11997
ZmHD-ZIP32	ZmHD-ZIP44	0.02278	0.04546	0.50109
ZmHD-ZIP33	ZmHD-ZIP37	0.07403	0.23353	0.31700
ZmHD-ZIP34	ZmHD-ZIP25	0.00737	0.02749	0.26809
ZmHD-ZIP35	ZmHD-ZIP09	0.02021	0.03196	0.63235
ZmHD-ZIP36	ZmHD-ZIP33	0.00993	0.04540	0.21872
ZmHD-ZIP39	ZmHD-ZIP32	0.02535	0.04556	0.55640
ZmHD-ZIP40	ZmHD-ZIP32	0.07258	0.23921	0.30341
ZmHD-ZIP41	ZmHD-ZIP47	0.00737	0.02749	0.26809
ZmHD-ZIP42	ZmHD-ZIP50	0.02021	0.03196	0.63235
ZmHD-ZIP43	ZmHD-ZIP36	0.00993	0.04540	0.21872
ZmHD-ZIP44	ZmHD-ZIP40	0.02535	0.04556	0.55640
ZmHD-ZIP45	ZmHD-ZIP09	0.07258	0.23921	0.30341
ZmHD-ZIP46	ZmHD-ZIP21	0.00227	0.03188	0.07120
ZmHD-ZIP47	ZmHD-ZIP22	0.00609	0.03630	0.16776
ZmHD-ZIP50	ZmHD-ZIP44	0.02408	0.04543	0.53004
ZmHD-ZIP51	ZmHD-ZIP43	0.07265	0.23342	0.31124

ZmHD-ZIP53	ZmHD-ZIP04	0.00987	0.03213	0.30718
ZmHD-ZIP54	ZmHD-ZIP03	0.00533	0.00737	0.72320
ZmHD-ZIP55	ZmHD-ZIP02	0.02408	0.03642	0.66117
ZmHD-ZIP56	ZmHD-ZIP01	0.00609	0.21284	0.02861
ZmHD-ZIP57	ZmHD-ZIP28	0.03527	0.07213	0.48897
ZmHD-ZIP59	ZmHD-ZIP16	0.05237	0.07096	0.73802
ZmHD-ZIP61	ZmHD-ZIP17	0.01709	0.03930	0.43486

Table S2. Primer sequences for gene cloning.

Primer name	Primer sequences (5’-3’)
ZmHD-ZIP02-F	ATGAGGCCAATGGCCAGCAATGGCA
ZmHD-ZIP02-R	GTGGAAGTGCTGGTGGTCGGCCCAC
ZmHD-ZIP05-F	ATGGATATTATGGCGCTTAATGCGA
ZmHD-ZIP05-R	CGGCCTCCGGGCTGGGCCGTCGACG
ZmHD-ZIP13-F	ATGAAGCCAATGGCCACCAATGGCA
ZmHD-ZIP13-R	CTGGAAGCAGGTGTGGTGTCCGTCG
ZmHD-ZIP14-F	ATGTCGACGGCGCTGGCGGTGGTCG
ZmHD-ZIP14-R	CACGAAGGACCAGTTCACGAACATG
ZmHD-ZIP16-F	ATGATGGAGAGGGTCGAGGACTTAG
ZmHD-ZIP16-R	CGTACTAGTGGCTCGGTCGATCGGG
ZmHD-ZIP23-F	ATGGACGGCGCCGAGGACGACGGGA
ZmHD-ZIP23-R	CTCGCTGAGGGACTCCAACTCCCAC
ZmHD-ZIP29-F	ATGGATCCGAGCGCGGTCAGTTTCG
ZmHD-ZIP29-R	GTTCCAGAACATGGATGTCCAGTTG
ZmHD-ZIP35-F	ATGAAGCCAATGGCAACCAATGGCA
ZmHD-ZIP35-R	CTGGAAGGCGTGGTGGCCGTCGGCC
ZmHD-ZIP42-F	ATGGCGCCTCAAAGCCTGGATCTCG
ZmHD-ZIP42-R	TCTGGCCGCCCTGAGGGCCTTTGCG
ZmHD-ZIP49-F	ATGGTGACGGCCAAGGAGGCCGCGG
ZmHD-ZIP49-R	CACAAACGACCAGTTGACGAACATG
ZmHD-ZIP53-F	ATGGCTCAGGAGGACGTCCACCTGG
ZmHD-ZIP53-R	GCAGGCGGCGGAGTGGGTGAAGGGG
ZmHD-ZIP55-F	ATGAGGCCAATGGCCAGCAATGGCA
ZmHD-ZIP55-R	ATGGAAGTGGTGGTGGTCGGCCCAC
ZmHD-ZIP58-F	ATGCCCGGGGGCCTGATGACCCCCG
ZmHD-ZIP58-R	ATGGTGGGACGTCGCCGCCGCCGCC
ZmHD-ZIP59-F	ATGGACAGGCCAGGCCACCACCAGC
ZmHD-ZIP59-R	GGCCACCGCGTTCCACTCCACAAGC
ZmHD-ZIP60-F	ATGGCAAATGCCGCTTGTGTGGGCG
ZmHD-ZIP60-R	ACAGACGTCACAGTGAAGTGCCGCC

Table S3. Primer sequences for PCR.

Primer name	Primer sequences (5'-3')
35S-F	ATTGATGTGATATCTCCACTGACGT
ZmHD-ZIP02-R	GTCCGACAGCTCGTCCTCGTTCACG
ZmHD-ZIP05-R	GCCAGGCTCCTCGTCGTCCTCGCTG
ZmHD-ZIP13-R	CTTGCGCTCGGGCTCCAGCTTGTTG
ZmHD-ZIP14-R	CTGACCACTTGTGACCACAGACTCA
ZmHD-ZIP16-R	GGGTGGTGGGCGGGGACATGTGCAT
ZmHD-ZIP23-R	GTCGTCCGCATTGCCGCCGTAGTAC
ZmHD-ZIP29-R	CATAGTCGGTCTCGAGTTGCTTGGT
ZmHD-ZIP35-R	GTGGGAGAGGAGGGCGTCGTTCTCG
ZmHD-ZIP42-R	AGGCACATTGTGAGGGCGGTGAGAG
ZmHD-ZIP49-R	GTCTCCTCCGCTATGGACATGAGCC
ZmHD-ZIP53-R	TTGAGGGTGCTGTGGTCCTTGAAGC
ZmHD-ZIP55-R	CTCCGCCTCCAGCTTGTTCCCGAGC
ZmHD-ZIP58-R	CTGGAACCAGAACTTGACCTGGAGA
ZmHD-ZIP59-R	CAGATCCTCCCGGTCTCTCAGCCTC
ZmHD-ZIP60-R	ATCGCTTCGAGCTGTTCGATCTGCC

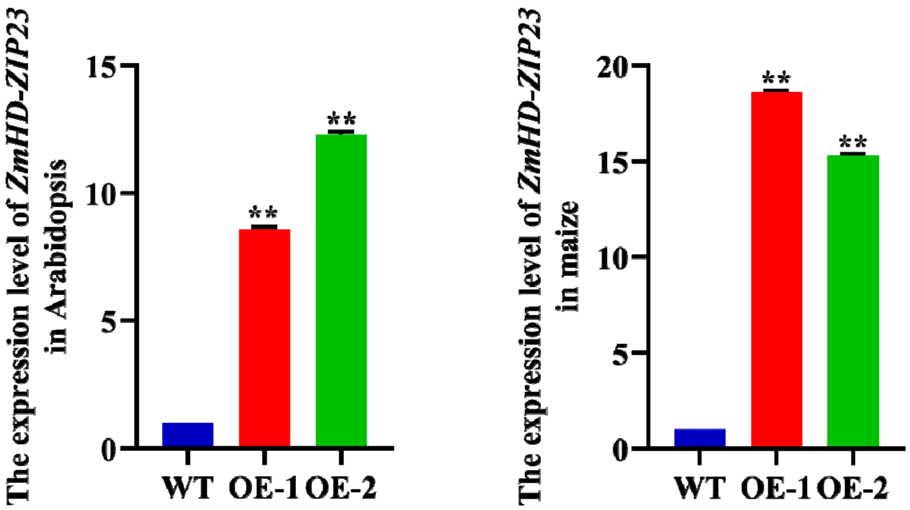

Figure S1. The expression level of *ZmHD-ZIP23* in Arabidopsis and maize
